# Immunomodulation of Myocardial Fibrosis

**DOI:** 10.1016/j.jacbts.2023.03.015

**Published:** 2023-06-14

**Authors:** Maurits A. Sikking, Sophie L.V.M. Stroeks, Federica Marelli-Berg, Stephane R.B. Heymans, Burkhard Ludewig, Job A.J. Verdonschot

**Affiliations:** a Department of Cardiology, Cardiovascular Research Institute Maastricht (CARIM), Maastricht University Medical Center (MUMC), Maastricht, the Netherlands; b William Harvey Research Institute, Queen Mary University of London, London, United Kingdom; c Department of Cardiovascular Research, University of Leuven, Leuven, Belgium; d Institute of Immunobiology, Kantonsspital St. Gallen, St. Gallen, Switzerland; e Department of Cardiology, University Heart Center, University Hospital Zurich, Zurich, Switzerland; f Department of Clinical Genetics, Maastricht University Medical Center (MUMC), Maastricht, the Netherlands

**Keywords:** cardiomyopathy, fibroblast, immune, immunology, immunotherapy, myocardial fibrosis

## Abstract

Immunotherapy is a potential cornerstone in the treatment of myocardial fibrosis. During a myocardial insult or heart failure, danger signals stimulate innate immune cells to produce chemokines and profibrotic cytokines, which initiate self-escalating inflammatory processes by attracting and stimulating adaptive immune cells. Stimulation of fibroblasts by inflammatory processes and the need to replace damaged cardiomyocytes fosters reshaping of the cardiac fibroblast landscape. In this review, we discuss new immunomodulatory strategies that manipulate and direct cardiac fibroblast activation and differentiation. In particular, we highlight immunomodulatory strategies that target fibroblasts such as chimeric antigen receptor T cells, interleukin-11, and invariant natural killer T-cells. Moreover, we discuss the potential of manipulating both innate and adaptive immune system components for the translation into clinical validation. Clearly, multiple pathways should be considered to develop innovative approaches to ameliorate myocardial fibrosis and hence to reduce the risk of heart failure.

## Highlights

• Myocardial fibrosis represents an unmet medical need, and its regulation by immunological mechanisms is increasingly evident.
• Emerging immunotherapies ameliorate myocardial fibrosis either by targeting cardiac fibroblast directly or via the immune system.
• Targeted immunotherapeutic approaches may reduce the risk of heart failure while mitigating potential safety concerns.

Myocardial damage may cause myocardial fibrosis, which ultimately predisposes the heart to cardiac arrhythmias and heart failure. Contrarily, myocardial fibrosis is also a process that is necessary to maintain the integrity of cardiac tissue after a myocardial insult (eg, infarction, viral infection, or cluster of differentiation [CD]4^+^ T-cell–mediated damage). A balance between adaptive and pathological myocardial fibrosis is thus necessary to maintain cardiac integrity because excessive fibrosis can lead to loss of cardiac function. The immune system offers an attractive platform to inhibit or even reverse pathological fibrotic remodeling of the heart.

**Figure 1:**
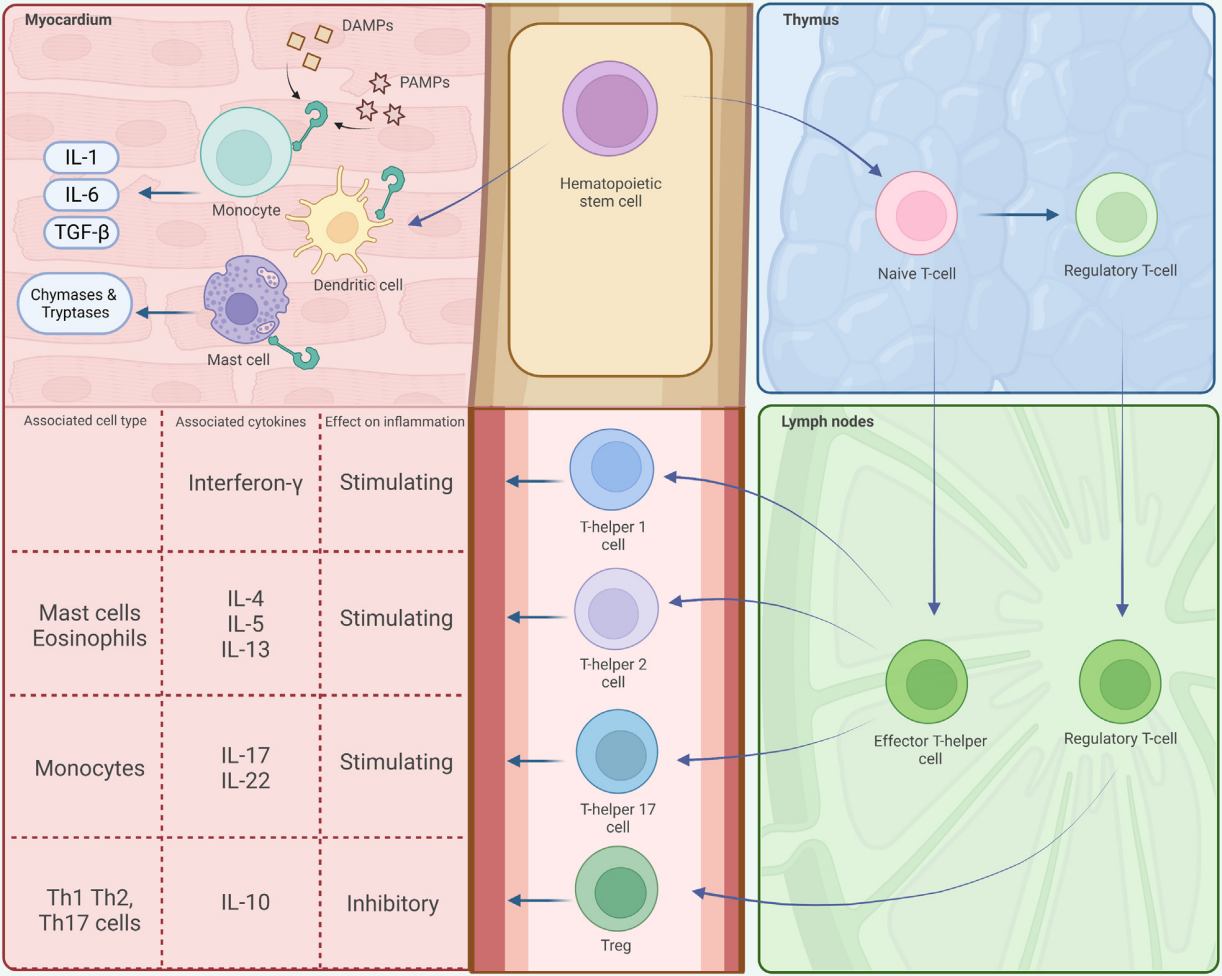
Overview of the Innate and Adaptive Immune System Immune cell progenitors are generated in the bone marrow. T cells further differentiate in the thymus, myeloid cells (dendritic cells, monocytes, mast cells, or neutrophils) migrate from the bone marrow via the blood into the myocardium. Mature T cells migrate from the thymus into lymphoid organs such as lymph nodes to receive differentiation signals that determine T-helper subset identity. Conserved molecular structures in tissues (danger-associated molecular patterns [DAMPs]) or derived from pathogens (pathogen-associated molecular patterns [PAMPs]) stimulate monocytes, dendritic cells, and mast cells to produce inflammatory and profibrotic cytokines. Mast cells also produce chymases and tryptases. Each T-cell subtype displays characteristic cytokine expression profiles and affects different target cell populations in the inflamed or fibrotic cardiac tissue. IL = interleukin; TGF = tumor growth factor.

The immune system plays a pivotal role as mediator between myocardial damage and fibrosis. Cardiomyocyte damage or death trigger danger signals that stimulate the innate immune system to release proinflammatory and profibrotic cytokines such as interleukin (IL)-1, IL-6, tumor necrosis factor (TNF)-α, and transforming growth factor (TGF)-β (Figure 1).^1^^,^^2^ Conversely, activated cardiomyocyte-specific T cells producing interferon-γ and IL-17 can trigger a cascading, cardiopathogenic immune response (Figure 1).^3^, ^4^, ^5^ In both processes, the interplay between innate and adaptive immune mechanisms foster profound changes in the cardiac fibroblast landscape leading to replacement of cardiomyocytes with fibrotic tissue (Central Illustration). Cardiac fibrosis represents thus, in the first place, a tissue reaction that is crucial for the maintenance of cardiac function. Excessive replacement of cardiomyocytes by larger fibrotic scars, however, negatively affects cardiac function due to adverse remodeling and stiffening of the ventricular wall. Thus, balancing and readjusting of fibrotic reactions in the myocardium bear the potential to reduce pathogenic myocardial fibrosis.

**Central Illustration:**
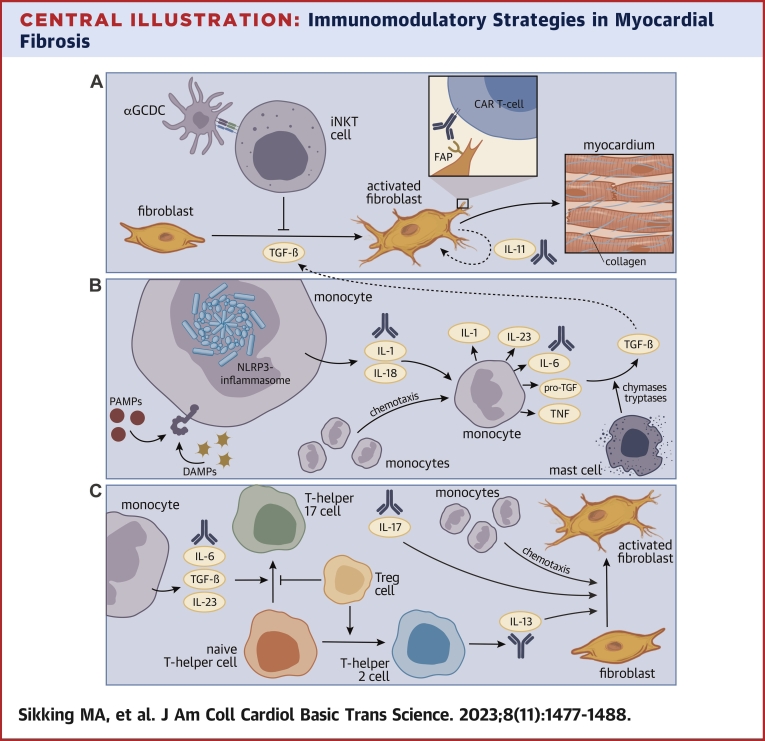
Immunomodulatory Strategies in Myocardial Fibrosis (A) Fibroblasts produce collagen and other extracellular matrix components. Fibroblasts are active by stimulation with TGF-β and other cytokines. IL-11 produced by activated fibroblasts creates a positive feedback loop by further increasing production of IL-11 and the extracellular matrix. Fibroblast activating protein (FAP)-specific CAR-T cells eliminate activated fibroblasts and anti-IL-11 therapy reduces fibroblast activation. α-Galactosylceramide (GCDC)-stimulated invariant natural killer T-cell (iNKT) cells downregulate the effects of TGF-β. (B) PAMPs and DAMPs activate the (nucleotide-binding domain leucine-rich-containing family pyrin domain-containing-3 [NLRP3]) inflammasome and lead to IL-1 production creating a positive feedback loop by increasing monocyte activation, IL-1, IL-6, and pro-TGF-β release. Dormant pro-TGF-β is enzymatically cleaved by chymases and tryptases into mature TGF-β. Anti-inflammasome or anti–IL-1 therapies reduce pro-TGF-β secretion. Lowering chymase or tryptase activity would reduce mature TGF-β formation. (C) Naive T cells differentiate into Th17 or Th2 cells depending on the nature and composition of costimulatory signals (eg, TGF-β and IL-6). IL-17 exhibits strong chemotactic properties on monocytes to migrate into the site of inflammation. IL-17 and IL-13 stimulates fibroblast activation and differentiation. Anti–IL-6 therapy could alter Th17 differentiation. Anti–IL-13 and anti–IL-17 therapies could alter fibroblast differentiation. CAR = chimeric antigen receptor; DAMP= damage-associated molecular pattern; FAP = fibroblast activation protein; IL = interleukin; iNKT = invariant natural killer T-cells; PAMP= pathogen-associated molecular pattern; TGF = tumor growth factor; Th cell = helper T cell; TNF = tumor necrosis factor; Treg = regulatory T cell.

In this review, we highlight different immunomodulatory strategies that could serve as means to ameliorate adverse cardiac remodeling. Promising approaches to counter myocardial fibrosis include: 1) immunomodulation of cardiac fibroblasts directly (Central Illustration A); 2) attenuation of specific innate immune responses (Central Illustration B); and 3) modulation of the adaptive immune system (Central Illustration C).

## Immunomodulation of Cardiac Fibroblasts

Cardiac fibroblasts are the key cellular component that determines the extent and quality of myocardial fibrosis. Fibroblasts include diverse subtypes based on embryonic origin, tissue location, and function, of which activity is controlled to some extent by proinflammatory cytokines (eg, TNF-α, IL-1, IL-17) that promote a phenotypical switch with altered extracellular matrix (ECM) production (Central Illustration A).^1^^,^^2^^,^^6^ Activated fibroblasts, in turn, produce chemokines that attract inflammatory cells and thereby create a positive feedback loop, which further increases inflammation and could potentiate fibrotic processes.^2^

### CAR T-cell therapy against activated fibroblasts

Chimeric antigen receptor (CAR) T-cell therapy is a novel therapy in oncology and is already approved for the treatment of melanoma patients.^7^ Such engineered T cells express recombinant antigen receptors, making it possible to induce apoptotic cell death in cells that express a targetable surface molecule. However, several challenges in CAR T-cell therapy remain such as the use of antigens that are exclusively expressed in the target tissue to prevent damage of cells in healthy tissues. A second major challenge in the application of CAR T cells for the treatment of myocardial fibrosis is the permanent CAR transcription, translation, and expression on the plasma membrane that occurs due to in vitro manufacturing of CAR T cells by implanting CAR DNA into the T-cell genome using a viral vector as DNA carrier. Nevertheless, recent advances have increased the interest in targeting cardiac fibrosis with CAR T-cell therapy.^8^^,^^9^ For example, lipid-nanoparticles can be targeted via anti-CD5 antibodies to T cells thereby facilitating mRNA-mediated transduction of CARs that detect fibroblast-activating protein (FAP) (Central Illustration A, Table 1). After uptake in T cells, mRNA can be released leading to the transcription of FAP-detecting CARs, which could alter fibrotic changes in hearts of mice with angiotensin-induced heart failure.^9^ However, due to the unstable nature of mRNA, the bioavailability of FAP CAR T cells appears to be only transient, and FAP-specific CAR T cells were undetectable at day 7 post-treatment.^9^ Most importantly, FAP CAR T cells are not specific for cardiac fibroblasts and could therefore elicit immunopathological damage in other organs.^10^ Additionally, the effect of FAP CAR T cells on cardiac fibroblast subtypes is not yet investigated.

**Table 1:** Mechanisms of Action and Limitations of Immunomodulatory Therapies

Immunomodulatory Therapy	Mechanism of Action	Limitations	Ref. #
CAR-T cells	Induction of apoptosis in activated fibroblasts	• Not heart specific • Negative effect on wound healing	^9^^,^^10^
IL-11 blockade	Modulation of fibroblast activation	• Function of IL-11 in tissue regeneration • Negative effect on wound healing	^13^^,^^18^
Inflammasome blockade (including colchicine)	Reduction of myocardial fibrosis	• Molecular mechanism not completely elucidated • Increased susceptibility to infections	27, 28, 29^,^^33^, ^34^, ^35^
Blocking chymases or tryptases	Modulation of fibroblast activation	• Molecular mechanism not completely elucidated • Safety data in humans are lacking	^39^^,^^50^^,^^52^
αGCDC-mediated iNKT cell therapy	Reduction of myocardial fibrosis	• Not heart specific. • No human data available.	^20^
IL-4 and IL-13 blockade	Modulation of fibroblast activation	• Unclear function in tissue regeneration • Negative effect on wound healing	^64^^,^^68^^,^^69^
IL-17 and IL-23 blockade	Modulation of fibroblast activation	• Cardiovascular adverse events after IL-23 blockade	^5^^,^^76^^,^^82^
Regulatory T-cell modulation	Improved tissue repair	• Molecular mechanism not completely elucidated	^59^^,^^91^^,^^93^

CAR = chimeric antigen receptor; IL = interleukin.

In sum, CAR T cells directed against FAP or other surface markers expressed by cardiomyocytes need to be carefully evaluated in preclinical models to ensure high specificity for the potentially damaging fibroblast population in the heart, while minimizing harmful side effects in healthy tissues.

### IL-11: a profibrotic cytokine released by fibroblasts

Recombinant human IL-11 was Food and Drug Administration approved in 1997 and marketed as Neumega (oprelvekin, Wyeth Pharmaceuticals) as the first platelet growth factor to treat severe thrombocytopenia or prevent severe thrombocytopenia following chemotherapy. Up to 15% of patients treated with Neumega developed atrial fibrillation in clinical trials.^11^ Additionally, elevated serum concentration of IL-11 is associated with cardiovascular events in patients with heart failure such as sudden cardiac death, myocardial infarction, and stroke.^12^ These clinical observations suggested that IL-11 could affect cardiac function. Indeed, the potential importance of IL-11 in cardiac fibrosis was revealed by transcriptomic analysis of human atrial tissue stimulated by TGF-β, which showed that *IL11* expression in fibroblasts is stringently regulated by TGF-β.^13^ In addition, in a preclinical model for profibrotic genetic cardiomyopathy, *Il11* expression was up-regulated in ECM-producing fibroblasts.^13^ It is possible that the effects of IL-11 in development of myocardial fibrosis involve further profibrotic pathways including endothelin-1, angiotensin II, PDGF, OSM, bFGF, TGF-β, and IL-13 (Central Illustration A, Table 1).^13^ Hence, it is likely that blockade of IL-11 (either in *Il11*-deficient mice or by IL-11–neutralizing antibodies), led to reduction in myocardial fibrosis and improvement of cardiac dysfunction through diminished ERK signaling and limited ECM production.^13^ Further studies have revealed antifibrotic and cardioprotective effects of IL-11 using recombinant human IL-11 instead of recombinant mouse IL-11 in mouse models of myocardial infarction,^14^ ischemia-reperfusion injury,^15^^,^^16^ and cold-ischemia.^17^ However, application of recombinant murine IL-11 in models of renal and cardiac fibrosis did not yield beneficial effects.^13^ These conflicting results could be due to the lack of effector functions of recombinant human IL-11 in mice or counter-regulation of murine IL-11 through the application of recombinant human IL-11. Clearly, further studies are warranted to resolve the function of IL-11 in cardiac fibroblast activation and differentiation including the potential of modulating fibroblastic microenvironments following myocardial damage.

Potential limitations of IL-11–based therapy of cardiac fibrosis are mainly based on the pleiotropic effects of this cytokine, and possibly the lack of knowledge on the effects of IL-11 blockade on fibroblast subtypes For example, Zebrafish lacking IL-11 signaling display severely compromised organ and tissue regeneration indicating that IL-11 exerts tissue-regenerative effects.^18^ The function of IL-11 in maintaining tissue functionality may be independent from profibrotic effects (eg, via STAT vs ERK signaling).^13^ Moreover, blocking of IL-11 in cardiac fibrosis should consider the important role of IL-11 in tissue repair.^18^

In sum, IL-11 is a new potential therapeutic target that may permit interference in fibrotic diseases, albeit with remaining specificity and safety concerns.

### Invariant natural killer T-cells

Invariant natural killer T-cells (iNKT cells) are an innate-like T-cell subset that express cell line–specific T cell receptor (*TCR*) gene segments not expressed by conventional T cells.^19^ These cells are activated by binding of their TCR to lipids presented by antigen-presenting cells in a CD1d-dependent manner during the acute phase of inflammation or infection without the necessity of previous immune training, hence their classification as innate-like cells.^19^

In a recent mouse study, Ikeda et al^20^ showed that numbers of splenic iNKT cells decrease during *Titin* mutation-induced dilated cardiomyopathy. When iNKT cells were activated by injecting dendritic cells presenting α-galactosylceramide on CD1d (αGCDC), the number of splenic iNKT cells increased.^20^ Subsequently, circulating interferon (IFN)-γ, IL-4, and IL-10 (released by iNKT cells) increased, in parallel with improved systolic dysfunction and reduced fibrotic gene expression compared with *Titin-*mutated mice treated with placebo.^20^ Only IFN-γ (and not IL-4 or IL-10) seemed to reduce fibrotic gene expression via Smad2/3 signaling, suggesting IFN-γ to be the main cytokine of iNKT cells to improve cardiac function of dilated cardiomyopathy mice.^20^

In sum, αGCDC infusion may stimulate iNKT cells to reduce myocardial fibrosis formation in dilated cardiomyopathy patients. A clinical trial to assess efficacy and safety of αGCDC in humans is already underway (Phase IIb Investigator-Initiated Multicenter Study of the Efficacy and Safety of Intravenous HUCV002-01 in Patients With Chronic Heart Failure; Japan Registry of Clinical Trials; jRCT2073210116).

## Attenuating Specific Innate Immune Responses

The innate immune system encompasses the host’s first line of defense against infections. Innate immune cells recognize danger signal (damage and pathogen associated patterns [DAMPs and PAMPs, respectively]) released by foreign invaders or by the host itself upon tissue damage (Figure 1).^1^ These PAMPs or DAMPs stimulate Toll-like receptor on the innate immune cell’s membrane leading to intracellular pathway activation (eg, the inflammasome-pathway) and extracellular release of proinflammatory cytokines such as TNF-α, IL-1, and IL-6 (Central Illustration B).^1^ Mast cells are part of the innate immune system that combats parasitic infections but also facilitate the activation step of dormant pro-TGF into active TGF (Central Illustration B).

### Inflammasome-dependent innate immune activation

The inflammasome is an intracellular danger receptor complex that is activated by conserved microbial molecules such a lipopolysaccharides (ie, PAMPs) or noninfectious, cell-derived distress signals (ie, DAMPS). The inflammasome complex is ubiquitously expressed, exerting functions in myeloid cells such as inflammatory monocytes and macrophages, as well as cardiomyocytes themselves.^21^^,^^22^ The inflammasome is constituted of an intracellular sensor protein (ie, a cytosolic pattern recognition receptor), its backbone structure (ie, an adaptor molecule), and the enzymatic effector domain (ie, a protease caspase-1). Activation of the inflammasome is initiated by a range of cytosolic pattern recognition receptors, which induce inflammasome complex assembly and hence signify distinct inflammasome pathways: nucleotide-binding domain leucine-rich repeat protein-3 (NLRP3) inflammasome, absent in melanoma-2 (AIM2) inflammasome, or pyrin inflammasome.^23^ After assembly, activated caspase-1 processes pro–IL-1β and pro–IL-18 into IL-1β and IL-18, respectively.^23^ Evidence that IL-1β directly stimulates fibrosis formation is scarce^24^; however, IL-1β seems to stimulate the inflammatory milieu using a positive feedback loop in which increasing IL-1β concentration amplifies IL-1β and IL-6 production, and thereby contributes to the maintenance of the proinflammatory state of fibroblasts during inflammatory reactions (Central Illustration B, Table 1).^1^^,^^24^

The NLRP3 inflammasome is by far the most studied inflammasome subtype.^25^ The classical activation of NLRP3 is a 2-step model comprising a priming and an activation signal.^26^ The priming signal may be a DAMP, a PAMP, TNF-α, or IL-1β (Central Illustration B). These signals increase expression of their cognate receptor (eg, Toll-like receptors or IL-1 receptor) stimulating nuclear factor kappa B (NF-κB) signaling and transcription of *NLRP3*, *IL1*, and *IL18* genes. Concomitant intracellular activation pathways involve ion influx (eg, calcium, potassium), or direct stimulation of the intracellular NLRP3 receptor. The breadth of NLRP3 activators (bacterial lipopolysaccharide, amyloid proteins, viral DNA, reactive oxygen species (ROS), oxalate crystals, pyrophosphate crystals, or urate crystals) indicates that the inflammasome act as general sensor of cellular distress.^26^ The major consequence of NLRP3 activation is the cleavage of pro–IL-1, pro–IL-18, and gasdermin D. Importantly, following cleavage, gasdermin D oligomers form pores in the plasma membrane through which IL-1 and IL-18 can leave the cell to bind to their receptors in neighboring cells or to signal back to the distressed cell in an autocrine fashion.^26^ Thus, inflammasome-dependent activation of the IL-1 pathway is a powerful, potentially self-amplifying innate immune activation pathway.

Following myocardial damage, the NLRP3 inflammasome and IL-18 have been shown to be involved in fibrotic changes of the myocardium induced by pressure overload, chronic angiotensin II stimulation, or acute β-adrenergic stimulation.^27^^,^^28^ However, the intricate molecular pathways and cellular interactions leading to myocardial remodeling due to inflammasome activation remain to be resolve (Table 1). For example, in 2 acute β-adrenergic stress models using isoproterenol as β-adrenergic stimulant, ROS stimulated the NLRP3 leading to elevated extracellular concentration of IL-18, but not IL-1.^27^^,^^28^ Blockade of either NLRP3 or IL-18 additionally reduced production of chemokines that attract monocytes, lowered expression of IL-6, with blunted proinflammatory macrophage infiltration and ameliorated myocardial fibrosis.^27^^,^^28^ Likewise, in situations of pressure overload–mediated cardiac damage, ROS and NF-κB stimulated NLRP3 inflammasome activation leading to increased IL-1 and IL-18 production and increased inflammatory monocyte infiltration and myocardial fibrosis.^29^^,^^30^ Similarly, NLRP3 inhibition has been shown to reduce inflammatory cell infiltration, lower IL-1 and IL-18 expression in fibroblasts, reduce myocardial fibrosis, and improve cardiac remodeling following myocardial infarction in mice,^31^^,^^32^ which suggested that the global inhibition of inflammasome activation could serve as a general approach to ameliorate cardiac damage following myocardial infarction.

In the CANTOS (Cardiovascular Risk Reduction Study [Reduction in Recurrent Major CV Disease Events]) trial, IL-1 blockade using canakinumab led to fatal infections and did not result in the improvement of all-cause mortality in myocardial infarction patients.^33^ It has been suggested that colchicine, a global inhibitor of intracellular protein transport, could function as an NLRP3 inhibitor and hence lower the risk of cardiovascular events. Interestingly, in 2 randomized controlled trials treating patients with myocardial infarction or chronic coronary disease with colchicine resulted in beneficial outcome defined as reduced cardiovascular death, myocardial infarction, ischemic stroke, and improved ischemia-driven coronary revascularization.^34^^,^^35^ Results of these studies are certainly encouraging, but should be interpreted with caution. First, the molecular mechanisms underlying the effects of colchicine on repair processes following myocardial damage are incompletely understood. Second, the serious side effects of colchicine potentially limit the applicability of this drug to a broad range of patients (Table 1).

In sum, inhibition of NLRP3 inflammasome and its downstream proinflammatory mediators IL-1 and IL-18 are interesting anti-inflammatory therapies with potential antifibrotic effects. However, patient selection is limited to inflammation-associated myocardial disease, as these therapies mainly down-regulate inflammation and the accompanying myocardial fibrosis.

### Modulation of the ECM by mast cells

Mast cells are cells of the myeloid cell family and are well-known mediators of allergic reactions, which are mediated by effector pathways that are required for the repulsion of parasitic infections. As key cellular component of the innate immune system, mast cells react against microorganisms or noninfectious stimuli due to their ability to recognize pathogen- or damage-associated patterns (Figure 1). Following activation, mast cells release different factors that exert either profibrotic and/or antifibrotic effects. Prestored modulators of ECM composition such as histamine, matrixmetalloproteinase-9, tryptases, chymase-1, cathepsin G, granzyme B, TNF-α, β-fibroblast growth factor (FGF), or TGF-β are stored in granules and released.^36^ Additionally, mast cells excrete cytokines such as IL-1, IL-3, IL-4, IL-5, or IL-8 that affect the cellular microenvironment.^36^^,^^37^ The intra- and extracellular regulation of these mediators of ECM and cell composition is complex, highly dependent on microenvironmental factors, and determines the phenotype of the mast cell as either profibrotic or antifibrotic.

Interestingly, patients with heart failure exhibit increased intracardiac mast cell infiltration when compared with healthy individuals.^38^ Such intracardiac accumulation of mast cell infiltration and degranulation correlated in some studies with myocardial fibrosis in patients with nonischemic and ischemic dilated cardiomyopathy and Chagas disease,^39^^,^^40^ findings that contrast earlier studies.^41^^,^^42^ It is possible that the activation status of mast cells within the cardiac environment determines to what extent ECM modification affects cardiac fibrosis. For example, particular sets of enzymes secreted by mast cells such as chymases and tryptases foster the induction of fibrosis through the activation of the dormant form of TGF-β. Cells release dormant TGF-β homodimers that are cleaved by chymases, tryptases, or other proteases enabling the TGF-β molecule to bind its receptor (Central Illustration B, Table 1).^43^, ^44^, ^45^ Moreover, chymases generate angiotensin I from angiotensin II,^46^^,^^47^ most likely leading to the generation of up to 75% of angiotensin II via the chymase pathway during heart failure.^48^ TGF-β and angiotensin II both stimulate fibroblast activation with increased production of collagen type I and III.^49^ Consequently, chymase inhibition can reduce myocardial fibrosis following myocardial infarction, myocardial ischemia, and during pacing-induced cardiomyopathy in rat, mouse, and canine models, respectively.^50^, ^51^, ^52^ Likewise, in vitro and in vivo mouse studies have shown that tryptases directly stimulate collagen production by cardiac fibroblasts.^53^, ^54^, ^55^ Intracardiac expression of tryptase is increased in mice with hypertension, overload cardiomyopathy, or following viral myocarditis.^53^^,^^56^^,^^57^ The inhibition of tryptase signaling, for example by protease activated receptor 2 blockade, prevented myocardial fibrosis in an hypertensive cardiomyopathy mice model,^55^ providing further evidence for cardioprotective effects through the manipulation of ECM composition. However, translation of these approaches into clinical practice is still in its infancy. Currently, only 1 phase II clinical trial has been registered to assess the safety of chymase inhibition following myocardial infarction (Table 1).^58^

In sum, mast cell inhibition including the modulation of ECM-modifying enzymes are promising avenues to counteract degenerative processes in the myocardium. Future translational studies should aim at establishing proof-of-concept for the suggested mode-of-action in clinical studies.

## Modulation of the Adaptive Immune System

The adaptive immune system is formed by specific antigen receptor-bearing B and T cells in which the latter are subdivided in cytotoxic, helper (Th cells), and regulatory T cells (Figure 1). Th cells are classified in 1, 2, and 17 cells (Th1, Th2, and Th17) (Figure 1). Such phenotypical classifications have to be taken with a grain of salt because of substantial function overlap between the cell types. For example, Th2 and Th17 cytokines can be expressed by the same cell.^59^^,^^60^ Nevertheless, it is the high potency and the adaptability of Th cells within the tissue environment that guarantee the efficient steering of immune responses by this particular immune cell subset.

### The Th2-mediated immune response

The T helper 2-type immune-axis has evolved to provide protection against (chronic) parasite infections (eg, helminth infections), but is presently (in developed and largely parasite-free countries) mostly known as the mediator of allergic disease. The Th2 cell is the signature cell type for allergy-associated inflammatory processes, which involve as well innate lymphoid type 2 cells (ILC2), eosinophils, and mast cells.^61^ Cytokines typically associated with the Th2 immune-axis are IL-4, IL-5, and IL-13 (Central Illustration C). Lately, interest in Th2-mediated immunity has expanded from allergic diseases to many other proinflammatory or profibrotic diseases. For an extensive review on Th2-mediated diseases and fibrosis, we refer to Gieseck et al.^61^

IL-13 induces tissue fibrosis indirectly by up-regulating TGF-β secretion and directly by stimulating the activation of monocytes and fibroblasts (Central Illustration C).^62^, ^63^, ^64^ IL-4 or IL-13 together with TNFα lead to the up-regulation of the IL-13 receptor on monocytes.^64^ Subsequent stimulation of this receptor activates the *TGFβ* promotor gene segment via activator protein-1 leading to TGF-β release from monocytes and subsequent stimulation of fibroblasts.^62^, ^63^, ^64^ Additionally, a hepatic fibrosis mouse model of chronic *Schistosoma mansoni* infection showed that IL-13 directly targets fibroblasts.^65^ Likewise, in a chronic angiotensin II stimulation mouse model, production of IL-4 and IL-13 by Th2 cells led to fibroblast activation and ECM formation.^62^

Interestingly, blockade of Th2 cell activation led to a delay of heart failure severity in mice undergoing transaortic constriction (TAC).^66^ Th2 cell activation was blocked using abatacept, a costimulatory signal blocker often used in rheumatoid arthritis.^67^ In this mouse model, TAC led to an intracardiac influx of Th2 cells measured 4 weeks after TAC.^66^ Abatacept led to an improved systolic function and reduced collagen formation, possibly due to its direct effect on Th2 because transcripts for Th1 and Th17 were not up-regulated.^66^ A second possible explanation for the antifibrotic effect of abatacept emphasized in the publication is the up-regulation of IL-10 produced by B-cells.^66^

Cardiac regeneration and repair is profoundly affected by chronic IL-4 or IL-13 blockade as shown in mice deficient for the respective Th2 cytokine (Table 1). Neonatal *Il4-* and *Il13-*deficient mice showed impaired cardiac function after myocardial infarction when compared with gene-proficient mice.^68^ Adult mice with *Il4* or *Il13* deficiency experienced tissue repair impairment following myocardial infarction as judged by reduced accumulation of reparative macrophages.^69^ Surprisingly, long-term postmarketing safety surveillance does not shown significant cardiovascular adverse effects in patients using chronic IL-4 or IL-13 blocking therapies for noncardiac indications.^70^^,^^71^

In sum, IL-4 and IL-13 are most likely involved in cardiac remodeling processes in experimental models. Future studies on immunophenotypic characteristics of myocardial fibrosis should look at Th2 fibroblast interactions to extend the evidence of the Th2-mediated fibrotic response.

### The Th17-mediated immune response

The Th17-mediated immune response combats extracellular bacteria and fungi. The Th17 cell is the predominant cell type involved in these processes, which involve as well the IL-17 production by natural killer cells, macrophages, and innate lymphoid cells.^72^ Hence, diseases that are triggered by bacterial products such as psoriasis and are associated with an excessive activation of Th17 cells can be treated through IL-17 blockade^73^). IL-6 and TGFβ stimulate the differentiation of naive Th cells into Th17 cells with ongoing stimulation with IL-23 being necessary for Th17 cells survival.^74^ Thus, IL-17 and IL-23 are potentially interesting therapeutic targets in Th17-mediated inflammatory diseases.

The IL-17 family consists of 6 members (IL-17A to IL-17F) and 5 receptors (IL-17RA to IL-17RE) in which IL-17A is the signature cytokine of Th17 cells.^72^ By itself, IL-17 is not a potent profibrotic cytokine, but exhibits potent chemotactic effects on neutrophils and monocytes to attract them to the site of inflammation, and converts monocytes into a profibrotic phenotype (Central Illustration C, Table 1).^75^^,^^76^ IL-17A seems to stimulate epithelial-to-mesenchymal transition, TGF-β secretion, and TGFβ receptor expression at the site of inflammation, a finding that has been mainly demonstrated in liver fibrosis.^76^, ^77^, ^78^ IL-17A stimulates fibroblasts to release granulocyte-monocyte colony stimulating factor (GM-CSF) leading to an influx of monocytes which in turn release IL-6, TGF-β, and IL-23 (Central Illustration C, Table 1).^76^^,^^79^^,^^80^ A positive feedback loop is triggered by IL-6– and TGF-β-stimulated differentiation of naive T cells into Th17 cells.^5^ Hence in this feedback loop, monocytes stimulate the formation of Th17 cells which in turn stimulate the influx of monocytes. TGF-β is continuously expressed by the monocytes and is a potent driver of myocardial fibrosis. A potential second driver of myocardial fibrosis in this feedback loop is GM-CSF.^80^ GM-CSF is released by activated fibroblasts and stimulates monocytes to express C-C chemokine ligand 17 (CCL17). Although exact mechanisms are not completely elucidated, deletion of *Ccl17* in mice seems to reduce myocardial fibrosis and improve cardiac function after myocardial infarction, infusion with angiotensin II or infusion with phenylephrine, possibly by reducing the influx of anti-inflammatory regulatory T cells (Tregs).^80^ Th17 cells and IL-17 may be central drivers of a complex interplay between Th17 cells, monocytes and fibroblasts in which IL-17 directly stimulates TGF-β release and indirectly GM-CSF release, ultimately leading to myocardial fibrosis.

Myocardial inflammation and progression of acute disease to chronic inflammatory cardiomyopathy is to some extent dependent on the presence of IL-17 signals as demonstrated in different murine models.^3^^,^^75^^,^^76^ Thus, myocardial fibrosis in chronic inflammatory cardiomyopathy is, at least partially, mediated by IL-17–dependent inflammation.^3^^,^^5^ This immunopathological principle was confirmed in a cohort of acute myocarditis patients, which in addition showed the dynamic biological interplay between IL-6, TGF-β, and IL-17 following acute myocardial inflammation.^5^ However, the confirmation of such complex cytokine interactions during chronic inflammatory cardiomyopathy still requires validation. Nevertheless, it is tempting to speculate that continuous myocardial damage mediated by persistent activation of cardiac myosin-specific and IL-17–producing Th cells^4^ precipitates cascading immune activation with recruitment of inflammatory macrophages and reprogramming of fibroblasts.

IL-23 is released by dendritic cells and monocytes upon (non)septic activation (Central Illustration B and C).^79^ Initial stimulation of naive T-cells by IL-6 and TGF-β leads to differentiation into Th17 cells and IL-23 receptor expression.^79^ Anti–IL-23 monoclonal antibodies down-regulate Th17-mediated immunity and should lower myocardial inflammation and fibrosis. However, IL-23 seems to foster down-tuning of Th1 immunity following myocardial infarction as shown in mice with permanent occlusion of the left anterior descending coronary artery that led to higher myocardial expression of interferon-γ, worse hemodynamic parameters, and worse survival.^81^ A pooled analysis of phase II, III, and an open label extension trial suggested increased risk of myocardial infarction and cardiovascular death in patients treated with briakinumab (an anti-IL12/23 monoclonal antibody) resulting in discontinued development of this drug (Table 1).^82^ Importantly, (postmarketing) safety analyses of other IL-23 blocking therapies suggest no increase in cardiovascular risk, for example, for patients with psoriasis or Crohn’s disease.^83^^,^^84^

In sum, IL-17 is a promising target to prevent Th17-mediated myocardial fibrosis, whereas therapeutic interventions based on IL-23 blockade require further safety testing.

### Regulatory T-cells

Tregs counter activation of inflammatory mechanisms in various tissues and therefore play a prominent role in the control of autoimmune reactions. The differentiation of naive T cells into Tregs is mediated by TGF-β with high concentrations of TGF-β favoring Treg formation, whereas low concentrations of TGF-β favor Th17 differentiation.^85^ Thus, Tregs acquire their particular phenotype depending on the milieu of the target tissue. Under conditions of tissue repair or during anti-inflammatory processes, Tregs express a profibrotic or anti-inflammatory phenotype, respectively.^86^^,^^87^ For anti-inflammatory effects, costimulatory signals drive Tregs predominantly into anti-Th1, anti-Th2, or anti-Th17 phenotype to calibrate particular anti-inflammatory effects.^88^^,^^89^ Such versatility in the acquisition of profibrotic or anti-inflammatory phenotypes may explain some of the contradicting functions that have been assigned to Tregs during myocardial fibrosis (Central Illustration C, Table 1).

In acute myocardial infarction, tissue damage may up-regulate IL-33 and CC chemokine 5 (CCR5) leading to accumulation of Tregs in the myocardial tissue.^87^^,^^90^^,^^91^ These Tregs produce amphiregulin and IL-10, and stimulate monocyte differentiation to support tissue repair.^91^^,^^92^ In chronic heart failure, Treg functions have been described to be highly variable with the acquisition of proinflammatory properties; noteworthy, evidence for proinflammatory Tregs is only established in post-myocardial infarction mouse models.^59^^,^^80^^,^^93^ Clearly, chronic heart failure patients show reduced abundance of circulating Tregs compared with patients with atherosclerosis or arrhythmias without heart failure.^93^ Moreover, a direct negative correlation has been demonstrated between circulating Tregs and heart failure progression with low circulating Treg frequency being associated with a higher risk of decompensated heart failure.^93^ Besides a decrease in circulating Tregs, a reduction of Treg migration into myocardial tissues was observed in mice in which heart failure was induced by myocardial infarction, angiotensin II infusion, or phenylephrine infusion.^80^ By deleting *Ccl17*, Treg migration into the myocardium improved, leading to lower myocardial fibrosis and improved cardiac function.

Targeting Tregs and/or improving their function in myocardial inflammation or fibrosis in patients is still a more theoretical option. So far, only 1 phase I/II clinical Treg trial is registered in which IL-2 is used to increase Treg cell numbers to assess safety of and efficacy of IL-2 to increase circulating Treg levels by 75% in patients with ischemic heart disease.^94^

In sum, Tregs stimulate tissue repair following acute myocardial infarction but lose their beneficial functions in the chronically injured heart, as shown in mouse models and heart failure patients. Future clinical trials may build on current preclinical data and concept to augment anti-inflammatory Treg functions in chronic heart failure.

## Conclusions and Future Directions

Current progress in immunomodulatory strategies of myocardial fibrosis is exciting, as reflected in the increasing number of publications on this topic. Potential therapies already reviewed recently^95^, ^96^, ^97^ and outside the scope of this review were immune-modulating exosomes and microRNAs, immunoadsorption and intravenous immunoglobulin, and rituximab.^98^, ^99^, ^100^ The safety profile of some therapies has already been evaluated (eg, colchicine, anakinra). For other therapeutic modalities, further extensive (pre)clinical testing is warranted (Table 1). Manipulating fibroblast activity in the inflamed or fibrotic cardiac microenvironment is a double-edged sword because excessive inhibition or augmentation of particular molecular or cellular pathways may worsen disease. Nevertheless, fibroblast activity is at the core of fibrotic processes in the myocardium and need therefore be considered as the prime target for interventional approaches. Direct or indirect modulation of fibroblast activity in the myocardium needs to be recognized as the key step in the treatment of myocardial diseases that are caused by excessive myocardial inflammation and fibrotic remodeling.

## Funding Support and Author Disclosures

This work was funded by the Dutch Cardiovascular Alliance, an initiative with support of the Dutch Heart Foundation, and Stichting Hartedroom for financing the Double Dose program 2020B005. Dr Marelli-Berg is supported by the British Heart Foundation (CH/15/2/32064, AA/18/5/34222). Dr Verdonschot is supported by a grant from the Dutch Heart Foundation–Dekker Clinical Scientist. All other authors have reported that they have no relationships relevant to the contents of this paper to disclose.
